# Small Molecule Drug Discovery at the Glucagon-Like Peptide-1 Receptor

**DOI:** 10.1155/2012/709893

**Published:** 2012-02-23

**Authors:** Francis S. Willard, Ana B. Bueno, Kyle W. Sloop

**Affiliations:** ^1^Translational Science and Technologies, Lilly Research Laboratories, Eli Lilly and Company, Indianapolis, IN 46285, USA; ^2^Centro de Investigación Lilly, Eli Lilly and Company, 28108 Alcobendas, Madrid, Spain; ^3^Endocrine Discovery, Lilly Research Laboratories, Eli Lilly and Company, Indianapolis, IN 46285, USA

## Abstract

The therapeutic success of peptide glucagon-like peptide-1 (GLP-1) receptor agonists for the treatment of type 2 diabetes mellitus has inspired discovery efforts aimed at developing orally available small molecule GLP-1 receptor agonists. Although the GLP-1 receptor is a member of the structurally complex class B1 family of GPCRs, in recent years, a diverse array of orthosteric and allosteric nonpeptide ligands has been reported. These compounds include antagonists, agonists, and positive allosteric modulators with intrinsic efficacy. In this paper, a comprehensive review of currently disclosed small molecule GLP-1 receptor ligands is presented. In addition, examples of “ligand bias” and “probe dependency” for the GLP-1 receptor are discussed; these emerging concepts may influence further optimization of known molecules or persuade designs of expanded screening strategies to identify novel chemical starting points for GLP-1 receptor drug discovery.

## 1. Introduction

The glucagon-like peptide-1 (GLP-1) receptor is a member of the peptide hormone binding class B1 (secretin-like receptors) family of seven transmembrane spanning, heterotrimeric G-protein coupled receptors (GPCRs). The best characterized physiologic role of the GLP-1 receptor is to help regulate insulin secretion from pancreatic *β* cells [1]. GLP-1 binding to the receptor activates G*α* *_s_*, stimulating membrane-associated adenylyl cyclases and cyclic 3′5′AMP (cAMP) production which enhances glucose dependent insulin secretion. The GLP-1 receptor peptide agonists, exenatide (exendin-4) and liraglutide, are widely approved medicines for the treatment of type 2 diabetes mellitus (T2DM) [2].

Identifying and developing small molecular weight organic compounds that mimic the orthosteric binding and receptor activation properties of GLP-1 peptide agonists is difficult. Class A GPCRs, for which many therapeutic small molecules have been developed [3], are structurally distinct from class B1 GPCRs. Class B1 receptors contain a larger independently folded globular ectodomain (ECD) at their N-termini. Peptide ligand binding to the ECD to initiate signaling of class B1 GPCRs is mechanistically different compared to class A receptors whose ligands primarily make contact with residues located within the membrane spanning *α*-helical regions [4]. For class B1 receptors, peptide ligands make numerous contacts with the ECD and extracellular loops of the transmembrane bundle [4]. For class A receptors, medicinal chemistry efforts have successfully exploited the endogenous ligand binding sites within transmembrane domains [3]. Recent reports solving X-ray crystal structures of class A GPCRs demonstrate the molecular interactions used for ligand binding [5–10].

While basic research efforts to better understand the intricate mechanisms regulating GLP-1 receptor function are being aided by advancements in GPCR molecular and structural biology (see review by Willard and Sloop in this issue of *Experimental Diabetes Research*), the field awaits determination of a high resolution crystal structure of a class B1 GPCR for use as a template to facilitate rational drug design for these difficult targets. In recent years, though, there have been an increasing number of reports showing discovery of structurally diverse small molecule ligands for the GLP-1 receptor. While the molecular details of compound-receptor binding are largely not determined, evidence supporting interaction of ligands with the GLP-1 receptor is provided in many cases. While several of these molecules only have utility as research tools, some may represent pharmacophores to be further optimized for clinical evaluation. Importantly, although not thought to be utilized endogenously to regulate GLP-1 receptor signaling, there does appear to be evidence generated for some scaffolds indicating the presence of an allosteric pocket(s) in the GLP-1 receptor [11–13], possibly located within the transmembrane domains. As a therapeutic strategy, small molecules targeting this pocket may be optimized to enhance binding and signaling of endogenous GLP-1 receptor peptides.

The development of orally available modulators of the GLP-1 receptor for therapeutic evaluation not only requires identification of specific nonpeptide ligands but also necessitates optimizing molecules to possess appropriate physicochemical properties. This is the medicinal chemistry concept of “drug-like” compounds, that is, molecules possessing functional groups and/or having properties consistent with the majority of known drugs [14]. Careful analyses of orally active, marketed drugs have resulted in several proposed rules for guiding optimization of key physical properties of compounds. Examples of these include the pioneering “rule of five” from Lipinski [15] (Rule of five: MW < 500 Da; log⁡ *P* < 5; number of hydrogen bond donors (HBD) < 5; number of hydrogen bond acceptors (HBA) < 10. Compounds that violate two or more of these rules have very low probability of being developed as an oral drug.) and properties identified by Veber et al. [16] (Polar surface area (PSA) ≤ 140; number of rotatable bonds ≤10. Compounds that meet these two criteria have high probability of achieving good oral bioavailability.). These guidelines are commonly used in medicinal chemistry strategies, and therefore, the drug-like profile of several GLP-1 receptor ligands is evaluated herein.

Below are descriptions of the best characterized small molecule GLP-1 receptor ligands. Although clinical development of any of these compounds is uncertain, the data suggest small molecules can be identified that target the GLP-1 receptor. In addition to descriptions of published GLP-1 receptor agonists, other chemotypes, including antagonists and molecules only reported in the patent literature that lack thorough biological characterization, are presented. The comprehensive dissemination of knowledge for small molecule ligands of this receptor may inspire advances in chemical and biological approaches for the GLP-1 receptor.

## 2. Low Molecular Weight GLP-1 Receptor Antagonists

### 2.1. PNU-126814

A small molecule GLP-1 receptor antagonist, PNU-126814, was disclosed in an abstract [17]. This compound is described to have submicromolar binding affinity for the GLP-1 receptor and inhibit GLP-1 induced cAMP modulation and insulin secretion in RINmF5 insulinoma cells. Unfortunately, the chemical structure of this compound has not been disclosed.

### 2.2. T-0632

**Figure 1:**
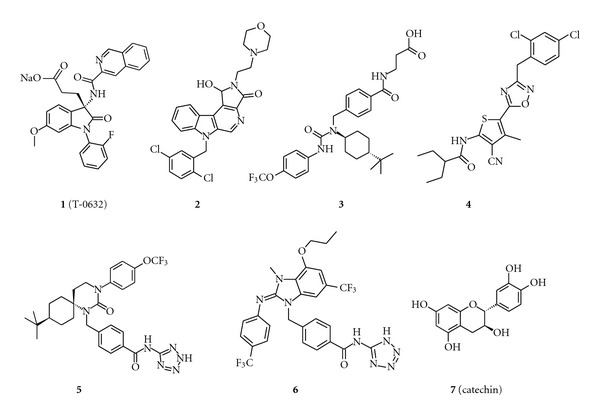
Chemical structures of GLP-1 receptor antagonists. Representative depictions of (**1**) T-0632, (**2**) 9-benzylpyrido[3,4-b]indole, (**3-6**) nonselective glucagon receptor antagonists, and (**7**) catechin.

The first small molecule modulator of the GLP-1 receptor to be described in the public domain with both annotated biology and chemistry is the antagonist T-0632. Beinborn et al. discovered that the cholecystokinin receptor 1 antagonist, T-0632 (Figure 1 (**1**) sodium (*S*)-3-[1-(2-fluorophenyl)-2,3-dihydro-3-[(3-isoquinolinyl-carbonyl)amino]-6-methoxy-2-oxo-1*H*-indole]propanoate), is a noncompetitive antagonist of the human GLP-1 receptor with low micromolar potency [18]. The binding site of this molecule is hypothesized to be in the ECD of the receptor as Trp^33^, within the ECD, was identified as a critical determinant for compound action. Thus, the Trp^33^Ser mutation in the human receptor results in a ~100-fold decrease in the binding affinity of the antagonist. Trp^33^ is within 10 Å of the peptide binding cleft in the crystal structures of GLP-1 and exendin-4 complexed with the GLP-1 receptor ECD [19, 20]. Trp^33^ does not make direct contact with the peptide ligands exendin-4 or GLP-1, but the data suggest that a small molecule binding event in this region of the protein could account for inhibitory activity. T-0632 can be considered an allosteric modulator of the GLP-1 receptor. Additionally, there is some evidence indicating the compound behaves as an inverse agonist in a constitutively active GLP-1 receptor system [21]. Although this compound could be a good tool for *in vivo* studies considering its physicochemical properties (it passes all of the Lipinski and Veber rules), the weak affinity of T-0632 for the GLP-1 receptor combined with its subnanomolar CCK1 antagonist activity renders it largely inadequate as a research tool to study the GLP-1 receptor.

### 2.3. 9-Benzylpyrido[3,4-b]Indoles

Agouron, Inc. described a family of molecules, exemplified by **2** (Figure 1 (**2**) 6-(2,5-dichlorobenzyl)-1-hydroxy-2-(2-morpholin-4-ylethyl)-1,6-dihydropyrrolo[3′,4′ : 5,6]pyrido[3,4-b]indol-3(2*H*)-one), as GLP-1 receptor antagonists [22]. Unfortunately, the patent describing these molecules does not include specific functional data, so the pharmacology of this chemotype of putative GLP-1 receptor antagonists remains to be elucidated. However, it is apparent that selective class B1 GPCR antagonists with well-defined pharmacology have utility in understanding biological systems and their sensitivity to therapeutic intervention [23].

### 2.4. Nonselective Glucagon Receptor Antagonists

A variety of weak GLP-1 receptor antagonists have been identified during investigations of glucagon receptor antagonists as potential therapeutic agents for T2DM. This finding suggests these two receptors may share a similar binding pocket. Figure 1 depicts representative molecules that bind both the GLP-1 and glucagon receptors. Molecules **3** to **6** (Figure 1 (**3**) *trans*-3-[[4-[[(4-*tert*-butylcyclohexyl)-(p-tolylcarbamoyl)amino]methyl]benzoyl]amino]propano-ic acid [24]; (**4**) *N*-[3-cyano-5-[3-[(2,4-dichlorophenyl)-methyl]-1,2,4-oxadiazol-5-yl]-4-methyl-2-thienyl]-2-ethyl-butanamide [25]; (**5**) *trans*-4-[[9-*tert*-butyl-2-oxo-3-(*p*-tolyl)-1,3-diazaspiro [5.5]undecan-1-yl]methyl]-*N*-(2*H*-tetrazol-5-yl)benzamide [26]; (**6**) 4-[[(2Z)-3,6-dimethyl-4-propoxy-2-(*p*-tolylimino)benzimidazol-1-yl]methyl]-*N*-(1*H*-tetrazol-5-yl)benzamide [27]) bind the GLP-1 receptor with affinities in the micromolar range.

A common characteristic of these compounds is high lipophilicity (calculated to be >5 for all four compounds) and molecular weight (around 500 Da). Unfortunately, it is not known whether such a high number of hydrophobes is required for GLP-1 receptor binding; focused optimization of these series against the GLP-1 receptor has not been reported. Importantly, the lipophilic nature of these molecules does not preclude achieving oral exposure as demonstrated by compound **6** in two animal species [27]. While all of these compounds are more potent glucagon receptor antagonists, and little is known about their respective structure activity relationships (SAR) against the GLP-1 receptor, some of the molecules could be attractive starting points for identifying potent and selective GLP-1 receptor ligands.

### 2.5. Catechin

The polyphenolic natural product, catechin (**7**) (Figure 1 (**7**) *trans*-2-(3,4-dihydroxyphenyl)chromane-3,5,7-triol), has been shown to be a functionally selective, negative allosteric modulator of the GLP-1 receptor [28]. This compound is further discussed in Section 4.

## 3. Low Molecular Weight GLP-1 Receptor Agonists

### 3.1. Quinoxalines

**Figure 2:**
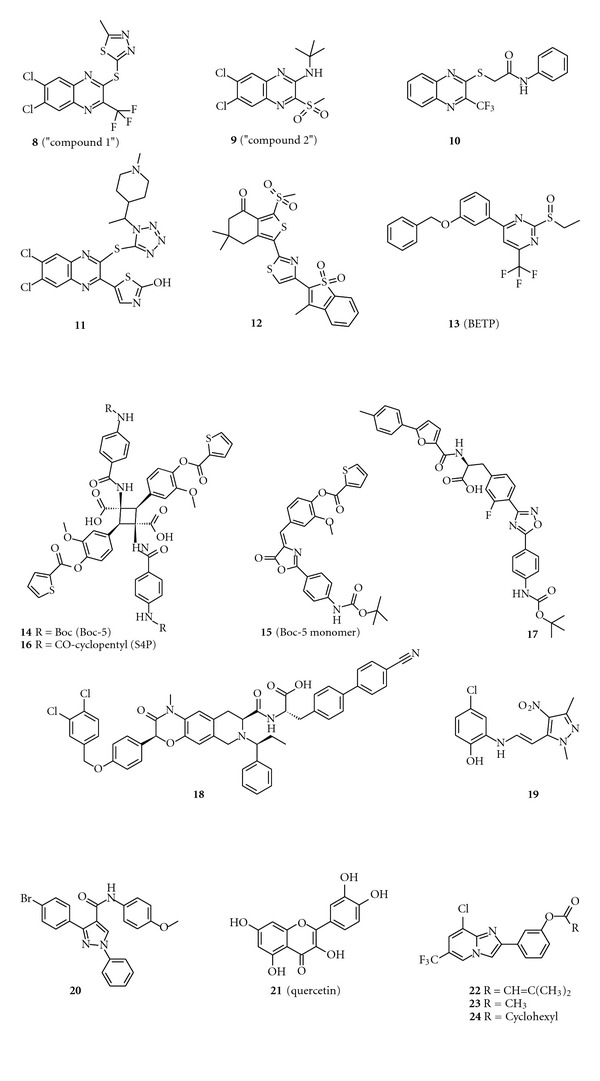
Chemical structures of GLP-1 receptor agonists. Representative depictions of (**8–11**) quinoxalines, (**12**) thiophene, (**13**) pyrimidine, (**14–16**) Boc-5 and derivatives, (**17**) phenylalanine, (**18**) azoanthracene, (**19**) pyrazole, (**20**) pyrazole-carboxamide, (**21**) flavonoid, and (**22–24**) imidazopyridines.

Teng, Knudsen et al. at Novo Nordisk disclosed a series of quinoxalines exemplified by **8** (Figure 2, (**8**) 2-[6,7-dichloro-3-(trifluoromethyl)quinoxalin-2-yl]sulfanyl-5-methyl-1,3,4-thiadiazole) (usually referred to in literature as “Compound 1”) and **9** (Figure 2, (**9**) N-*tert*-butyl-6,7-dichloro-3-methylsulfonyl-quinoxalin-2-amine) (usually referred to in literature as “Compound 2”) [12, 29]. Initial screening using a competitive binding assay did not provide useful hits from ~500,000 compounds. A change in strategy to perform screening using a functional assay led to the identification of the quinoxaline scaffold from ~250,000 compounds. Compound **9** is a full agonist in GLP-1 receptor dependent cAMP accumulation experiments and shows specificity for the GLP-1 receptor versus other class B1 GPCRs. Compound **9** is characterized as an ago-allosteric modulator of the GLP-1 receptor; it displays intrinsic activity and also enhances binding of GLP-1 to the GLP-1 receptor [12]. Moreover, compound **9** action is not blocked by exendin-4_(9-39)_, further supporting an allosteric mechanism of action of this molecule. Additional studies clearly demonstrate that compound **9** increases the binding affinity of both GLP-1 and oxyntomodulin for the GLP-1 receptor [11]. Together, these data are important because the results indicate the compound interacts with a site independent of the orthosteric binding pocket, suggesting the existence of an exploitable allosteric site for small molecules.

The definitive experiment to show compound **9** is a *bona fide* GLP-1 receptor ligand is the demonstration that it significantly potentiates glucose dependent insulin secretion in wild type mouse islets but not in islets from GLP-1 receptor knockout mice [12]. A subsequent report shows intraperitoneal administration of **9 **is insulinotropic and enhances glucose disposal in a glucose tolerance test [30].

Following the disclosure of compounds **8** and **9**, further SAR around the quinoxalines was conducted [31]. A sulfone, sulfoxide, or thioether in position 2 of the bicycle is essential for activity, and quinoxalines are superior to other heterocycles as GLP-1 receptor agonists. The investigation also shows the need for electron-withdrawing groups on the quinoxaline with 6,7-dichloroquinoxaline being the best core. It is noted in the SAR describing the optimization of compound **9** that the quinoxaline analogs are chemically unstable in the presence of nucleophiles and have high microsomal metabolism. It can be speculated that the labile sulfur-containing side chain is responsible for the instability of these compounds to nucleophiles. The poor chemical stability precludes longer-term *in vivo* studies with this compound.

There have been other efforts aimed at exploring this scaffold. Scientists from the New England Medical Center claimed a series of 2-thiosubstituted quinoxalines, represented by compound **10** (Figure 2 (**10**) 2-(3-methylquinoxalin-2-yl)sulfanyl-*N*-phenyl-acetamide), as weak GLP-1 receptor agonists [21]. Zydus also studied analogs of compound **8** but did not show improved activity over this compound in glucose dependent insulin secretion assays using RINmF5 insulinoma cells [32]. Recent reports from the Dong-A Pharmaceutical Company [33, 34] describe identification of a new series of 2-thioquinoxaline analogs of **8**. Compound **11** (Figure 2 (**11**) (5-[6,7-dichloro-3-[1-[1-(1-methyl-4-piperidyl)ethyl]tetrazol-5-yl]sulfanyl-quinoxalin-2-yl]thiazol-2-ol), disclosed as a racemic mixture, has 100 nM potency in a cAMP response element (CRE)-luciferase reporter assay. This molecule stimulates insulin secretion in INS-1E insulinoma cells and is selective against other class B1 GPCRs. Importantly, oral dosing of this compound enhances insulin secretion in a mouse intravenous glucose tolerance test model [33, 34]. It appears the Dong-A compound may overcome some of the instability issues observed with the initial quinoxalines to achieve effective oral exposure. 

### 3.2. Thiophenes

Besides the quinoxaline compounds, Novo Nordisk also disclosed a second family of GLP-1 receptor agonists, a series of sulfonyl-thiophenes represented by compound **12** (Figure 2) [35]. No biological data are provided, and these compounds share a common feature with the quinoxalines: a sulfonyl group attached to an aromatic ring. While in this case, the thiophene is an electron-rich ring and thus less prone to nucleophilic attack, one or two strong electron-withdrawing carbonyl groups occur in all of the examples, leading to speculation that the sulfonyl group in these systems is also labile.

### 3.3. Pyrimidines

Our group identified a series of pyrimidine based ago-allosteric modulators of the GLP-1 receptor exemplified by racemic compound **13** (Figure 2, (**13**) 4-(3-benzyloxyphenyl)-2-ethylsulfinyl-6-(trifluoromethyl)pyrimidine; BETP) [13]. The parent molecule of this series was found by screening a small library, generated from three-dimensional pharmacophore models, using HEK293 cells stably cotransfected with the human GLP-1 receptor and a CRE-luciferase reporter. Compound **13** shows GLP-1 receptor dependent activity in both CRE-luciferase and cAMP accumulation assays in HEK293 cells, and the molecule stimulates glucose dependent insulin secretion in *ex vivo* assays of both rodent and human islet preparations. In combination experiments with GLP-1, compound **13** is not competitive with ^125^I-GLP-1 but can act in an additive manner to enhance GLP-1 induced cAMP signaling and insulin secretion. Consistent with these findings, and similar to the quinoxalines, compound **13** action is not blocked by exendin-4_(9-39)_. Importantly, our studies show compound **13** induces insulin secretion *in vivo*. The molecule is active in animals undergoing either the intravenous glucose tolerance test or the hyperglycemic clamp assay. While these *in vivo *results are encouraging, significant improvement in various metabolic liabilities of this compound are necessary before longer term studies can be explored. Although compound **13** is stable upon incubation in plasma, it shows chemical instability in the presence of nucleophiles.

### 3.4. Boc-5

A significant advance in the development of small molecule GLP-1 receptor agonists is the discovery of substituted cyclobutanes, exemplified by compound **14** (known as Boc-5) (Figure 2 (**14**) 1,3-bis [[4-(*tert*-butoxycarbonylamino)benzoyl]amino]-2,4-bis [3-methoxy-4-(thiophene-2-carbonyloxy)phenyl]cyclobutane-1,3-dicarboxylic acid). Boc-5 was discovered using a high throughput CRE-luciferase screen for activators of the rat GLP-1 receptor [36]. Serendipity played a role in this discovery as the original compound selected for testing was an olefin that is half of the size of Boc-5. It was soon realized that the olefins (depicted as compound **15**, monomer of Boc-5) dimerize in the DMSO solution to become cyclobutanes, represented by Boc-5 and S4P (compound **16**), that are the real actives. While lacking structural characteristics of drug-like molecules (Boc-5 violates all of the Lipinsky and Veber rules), Boc-5 provides a useful proof of concept molecule for nonpeptide GLP-1 receptor agonists. Pharmacologically, Boc-5 is a full agonist in the CRE-luciferase assay, while the closely related molecule S4P is a partial agonist. In a cAMP accumulation assay, however, both Boc-5 and S4P are partial agonists. Importantly, both S4P and Boc-5 are functionally antagonized by exendin-4_(9–39)_ and displace ^125^I-GLP-1 in receptor binding assays. Inhibition of ^125^I-GLP-1 binding does not appear saturable [36], possibly suggesting an allosteric binding mechanism; however, this has yet to be elucidated.

Boc-5 dose dependently stimulates glucose dependent insulin secretion in isolated rat islets [36]. Paradoxically, 10 *μ*M of Boc-5 is more efficacious than a saturating concentration of GLP-1 in inducing insulin secretion. It is worth noting that these studies were performed at an abnormally high glucose concentration of 25 mM. Boc-5 is reported to have a plasma half life of approximately 8 hours following intraperitoneal dosing of the compound. Acute administration of Boc-5 shows an anorectic effect in C57/B6 mice that lasts over 12 hours in the intraperitoneally dosed group and 90 minutes in orally administered animals [36]. Although no exposure is reported for the oral study, the large difference in food intake observed in oral versus intraperitoneal administration probably indicates poor oral bioavailability of the compound. Chronic intraperitoneal administration to *db*/*db* mice lowers HbA1c, reduces food intake, lowers body weight, and enhances insulin secretion and glucose excursion (using an intraperitoneal glucose tolerance test) [36]. Importantly, acute effects of Boc-5 are entirely abrogated by coadministration of exendin-4_(9–39)_, suggesting a GLP-1 receptor dependent action [36]. Follow-up studies confirmed many of these findings using a diet-induced obesity mouse model [37]. A limited SAR around Boc-5 has been reported despite synthetic challenges of these molecules [38]. While some improvement in potency is shown, the new molecules share the poor physicochemical properties with S4P and Boc-5, which presumably preclude these compounds from being oral drugs.

### 3.5. Phenylalanines

Argusina, Inc. disclosed phenylalanine derivatives as GLP-1 receptor modulators, represented by compound **17**, (Figure 2 (**17**) 3-[4-[5-[4-(tert-butoxycarbonylamino)phenyl]-1,2,4-oxadiazol-3-yl]-3-fluoro-phenyl]-2-[[5-(*p*-tolyl)furan-2-carbonyl]amino]propanoic acid) [39]. The molecules are disclosed as racemic mixtures. One can envision the Argusina compounds as an optimization of the monomer of Boc-5. Thorough pharmacological evaluation of these compounds has not been disclosed, although the initial report suggests that compounds can modulate cAMP mobilization and insulin secretion in cell culture systems [39]. From a pharmacokinetic perspective, these molecules violate the Lipinski and Veber rules (large molecular weight and high lipophilicity; high PSA), so an improvement of the physicochemical properties of the scaffold would likely be required to achieve an orally active agent.

### 3.6. Azoanthracenes

Several patent disclosures from Transtech Pharmaceuticals (TTP) report identification of a number of azoanthracene and oxadiazoanthracene derivatives as GLP-1 receptor agonists [40–43], exemplified by compound **18**, (Figure 2 (**18**) (*S*)-3-(4′-cyano-biphenyl-4-yl)-2-{[(3*S*,7*S*)-3-[4-(3,4-dichloro-benzyloxy)-phenyl]-1-methyl-2-oxo-6-((*S*)-1-phenyl-propyl)-2,3,5,6,7,8-hexahydro-1*H*-4-oxa-1,6-diaza-anthracene-7-carbonyl]-amino}propionic acid) [43]. Molecules described in these disclosures have nanomolar potencies for the GLP-1 receptor in recombinant cell assays of cAMP, and there is some indication from the data that the compounds may be partial agonists [42]. A recent publication disclosed that TTP molecules are effective antidiabetic agents in preclinical rodent models of T2DM, stimulating glucose dependent insulin secretion in rodent islets and improving glucose excursion in an oral glucose tolerance test [44]. The leading molecule in this class, TTP054 (structure not disclosed) is reported to currently be under evaluation in Phase II clinical trials as an oral drug [44]. All of the molecules disclosed by TTP in these patents are of large molecular weight and high lipophilicity (**18**: MW ~ 880 Da, calculated log *P* = 10, 13 rotatable bonds) compared to typical orally administered medications, suggesting high doses of the compound would likely be required to achieve efficacious exposure.

### 3.7. Pyrazoles

A series of pyrazoles represented by compound **19** (Figure 2 (**19**) 4-chloro-2-[[(*E)*-2-(2,5-dimethyl-4-nitro-pyrazol-3-yl)vinyl]amino]phenol) were identified by Kopin and Beinborn as weak agonists of the GLP-1 receptor [21]. The compound has micromolar activity for the GLP-1 receptor in recombinant cell assays of cAMP [21]. No other studies have been disclosed with this kind of molecule.

### 3.8. Pyrazole-Carboxamides

A recent publication reported discovery of small molecule GLP-1 receptor potentiators as an ancillary outcome of efforts to identify glucagon receptor antagonists [45]. The authors used virtual screening of a library of commercially available drug-like compounds to search for compounds with physicochemical similarities to known glucagon receptor antagonists. This was followed by a homology model based docking approach. Compound **20**, (Figure 2 (**20**) 3-(4-bromophenyl)-*N*-(4-methoxyphenyl)-1-phenyl-pyrazole-4-carboxamide), identified as a potential candidate for glucagon receptor antagonism, does not show functional activity at this receptor, but it is observed to potentiate an EC_20_ concentration of GLP-1 induced cAMP production in TC6 cells [45]. The authors claim to have discovered a novel small molecule chemotype that potentiates the GLP-1 receptor. If these data are confirmed in recombinant cell systems where GLP-1 receptor dependence can be more definitively ascribed, this would be a breakthrough discovery with respect to GLP-1 receptor allosteric modulators. The ability to significantly potentiate either the affinity or efficacy (the data in this report do not distinguish between these possibilities) of GLP-1 would be highly desirable characteristics of a GLP-1 receptor targeted small molecule. Moreover, this would represent an important computational and operational approach to discovering class B1 GPCR positive allosteric modulators.

### 3.9. Flavonoids

Sexton et al. characterized a series of quercetin-like flavonoids, represented by quercetin (Figure 2 (**21**) 2-(3,4-dihydroxyphenyl)-3,5,7-trihydroxy-chromen-4-one), with positive allosteric modulator activity on the GLP-1 receptor [28, 46]. These compounds were originally identified by Domain Therapeutics as GLP-1 receptor modulators [46]. A thorough analysis of this chemical scaffold demonstrates this class of molecules positively modulates the affinity and efficacy of GLP-1 receptor peptide ligands. The quercetin series appears to show ligand bias in that it is specific for GLP-1 receptor mediated Ca^2+^ mobilization but not cAMP accumulation. Typical of polyphenolic compounds, a lack of robust functional effects, polypharmacology, and flat SAR limits the usefulness of this chemotype to *in vitro *studies and precludes optimization for pharmacological purposes. However, the discovery and analysis of this series of molecules represent a further proof of concept for allosteric modulators of the GLP-1 receptor.

### 3.10. Imidazopyridines

There is one published report describing a series of imidazopyridine based molecules. The initial hit, compound **22 **(Figure 2 (**22**) [3-(8-chloro-6-methyl-imidazo [1,2-a]pyridin-2-yl)phenyl] 3-methylbut-2-enoate), was identified from a library of 10,000 compounds using a GLP-1 receptor functional assay. The strategy used for optimization was to design a pharmacophore model from three known agonists (compounds **9** and **12** and the monomer of S4P, a close analog of monomer **15**), although there is no evidence of structural similarity between these three series. Nevertheless, analogs of compound **22** that fit additional features of the pharmacophore were designed.

Both compounds **23 **and **24**, (Figure 2 (**23**) 3-[8-chloro-6-(trifluoromethyl)imidazo [1,2-a]pyridin-2-yl]phenyl] acetate and (**24**) [3-[8-chloro-6-(trifluoromethyl)imidazo [1,2-a]pyridin-2-yl]phenyl] cyclohexanecarboxylate), show induction of GLP-1 receptor signaling in GLP-1 receptor expressing CHO or HEK293 cells [47]. These molecules are shown to have some agonist activity in heterologous cell lines expressing the GLP-1 receptor, but further investigation in other systems is not reported. While these are very small molecules, and pass the Lipinski and Veber rules, further optimization of the activity of the compounds against the GLP-1 receptor, and SAR work to move away from the labile ester of the phenol, is likely necessary for these compounds to become useful for oral studies.

## 4. Ligand Biased Signaling

Studies to more fully characterize several of the small molecule ligands of the GLP-1 receptor are needed to advance the field. An emerging concept in GPCR pharmacology is that of functional selectivity (also known as ligand bias or stimulus bias) [48, 49]. It is now appreciated that GPCR ligands can stabilize distinct receptor conformations, which in some instances, lead to differential modulation of signal transduction pathways [48]. To date, there is a single study reporting that the weak GLP-1 receptor peptide agonists, oxyntomodulin and glucagon, are biased toward G*α* *_s_* over *β*-arrestin for coupling to the GLP-1 receptor [50]. These peptides show low potency and partial agonism (>50% of GLP-1 efficacy) for *β*-arrestin recruitment at the GLP-1 receptor yet are full agonists for cAMP accumulation. In support of this, another report describes oxyntomodulin as having stimulus bias for the ERK1/2 activation pathway relative to cAMP or Ca^2+^ mobilization [11]. Further work is necessary to understand whether the observed *in vitro* functional selectivity of the various GLP-1 receptor peptide ligands is physiologically relevant *in vivo*. Such studies may be complicated by the polypharmacology of oxyntomodulin as it is a dual agonist of both glucagon and GLP-1 receptors and by receptor reserve phenomena that often lead to the classification of partial agonists as biased ligands [51].

Sexton et al. demonstrated two GLP-1 receptor ligands are functionally selective. Quercetin (**21**) and a subset of related naturally occurring flavonoids exhibit functional selectivity as these molecules potentiate GLP-1 peptide signaling in Ca^2+^ mobilization but not cAMP production [11, 28]. Similarly, the flavonoids also display probe dependence in that the molecules modulate both GLP-1 and exendin-4 action but not oxyntomodulin signal transduction. While a variety of flavonoid cores were tested in these studies, only the 3-hydroxyflavone core displayed functional activity. Unfortunately, flavonoids exert multiple pharmacologic effects at the concentrations required for the activation of the GLP-1 receptor. Thus, these are not good starting points for the identification of more potent allosteric ligands.

An SAR analysis of related compounds identified the polyphenolic natural product, catechin (**7**), as a probe dependent, functionally selective, and negative allosteric modulator of the GLP-1 receptor [28]. Both *trans* enantiomers of this compound are known as catechin, but it is not clear from this publication if the racemic compound or one *trans* enantiomer was used in this study. Catechin decreases the efficacy of GLP-1 signaling via cAMP but does not modulate non-cAMP signaling by the GLP-1 receptor peptide agonists nor does it significantly alter the pharmacology of exendin-4 or oxyntomodulin signaling. Analogously, the small molecule quinoxaline **8** [12] displays a complex profile of activity consistent with functional selectivity and probe dependence. Compound **8** shows affinity driven positive allosteric modulator activity for oxyntomodulin, and to a lesser extent, GLP-1 but not toward exendin-4. This activity is only observed for the cAMP pathway and not for the Ca^2+^ or ERK pathways, demonstrating probe dependence whereby allosteric modulation of the GLP-1 receptor is dependent on the species of the bound orthosteric agonist.

These studies are seminal as they provide proof of concept that allosteric modulation of the GLP-1 receptor can engender pathway specific modulation of signal transduction outcomes. It would be informative to identify small molecule ligands of the GLP-1 receptor with biased signaling to delineate the *in vivo *consequences of selective modulation of specific molecular signal transduction mechanisms. Importantly, this would provide a better understanding of the molecular mechanisms of the antidiabetic effects of GLP-1 receptor agonism. At this time, these *in vitro* studies demonstrate that probe dependence of allosteric modulators can occur at the GLP-1 receptor and identify critical aspects to be considered when optimizing small molecule GLP-1 receptor agonists or modulators. Future efforts also should be directed at mapping the structural determinants of allosteric ligand binding and at evaluating interaction between allosteric and orthosteric sites.

## 5. Conclusions

Despite 20 years of research following the molecular identification and cloning of the GLP-1 receptor [52, 53], no orally available small molecule GLP-1 receptor activator has been developed for therapeutic use. Encouragingly, however, the pace of identifying small molecule GLP-1 receptor ligands is increasing. In addition to the molecules discussed herein, the field anxiously awaits disclosures from both Addex Pharmaceuticals S.A. and Vivia Biotech S.L. that have recently presented data from their small molecule GLP-1 receptor modulator programs (see Cambridge Healthtech Institute, Discovery On Target 2011, Cambridge, MA, USA, Nov 2–4, 2011). Although the mechanisms of peptide binding and receptor activation of the GLP-1 receptor are complex and likely difficult to mimic with low molecular weight compounds, examples of small molecules working via an allosteric mode provide compelling evidence that medicinal chemistry strategies for this target should be considered. Further, the application of advanced structural biology methodologies and more sophisticated assay systems and testing schemes, including work to understand biased signaling for GLP-1 receptor ligands, will likely be needed to advance drug-like molecules.
